# *E. coli* K-12 and EHEC Genes Regulated by SdiA

**DOI:** 10.1371/journal.pone.0008946

**Published:** 2010-01-28

**Authors:** Jessica L. Dyszel, Jitesh A. Soares, Matthew C. Swearingen, Amber Lindsay, Jenee N. Smith, Brian M. M. Ahmer

**Affiliations:** Department of Microbiology, The Ohio State University, Columbus, Ohio, United States of America; University of Massachusetts Medical School, United States of America

## Abstract

**Background:**

*Escherichia* and *Salmonella* encode SdiA, a transcription factor of the LuxR family that regulates genes in response to *N*-acyl homoserine lactones (AHLs) produced by other species of bacteria. *E. coli* genes that change expression in the presence of plasmid-encoded *sdiA* have been identified by several labs. However, many of these genes were identified by overexpressing *sdiA* on a plasmid and have not been tested for a response to *sdiA* produced from its natural position in the chromosome or for a response to AHL.

**Methodology/Principal Findings:**

We determined that two important loci reported to respond to plasmid-based *sdiA*, *ftsQAZ* and *acrAB*, do not respond to *sdiA* expressed from its natural position in the chromosome or to AHLs. To identify genes that are regulated by chromosomal *sdiA* and/or AHLs, we screened 10,000 random transposon-based luciferase fusions in *E. coli* K-12 and a further 10,000 in *E. coli* O157:H7 for a response to AHL and then tested these genes for *sdiA*-dependence. We found that genes encoding the glutamate-dependent acid resistance system are up-regulated, and *fliE* is down-regulated, by *sdiA*. Gene regulation by *sdiA* of *E. coli* is only partially dependent upon AHL.

**Conclusions/Significance:**

The genes of *E. coli* that respond to plasmid-based expression of *sdiA* are largely different than those that respond to chromosomal *sdiA* and/or AHL. This has significant implications for determining the true function of AHL detection by *E. coli*.

## Introduction

Prokaryotes have the ability to coordinate their gene regulation and behavior in response to population density, effectively acting as multicellular organisms. The detection of population density is referred to as quorum sensing [1], [2]. A common mechanism of quorum sensing among the gram-negative bacteria is the synthesis and detection of a diffusible molecule of the *N*-acyl homoserine lactone (AHL) type (reviewed in [3], [4], [5]). The prototypical example is the regulation of bioluminescence by *Vibrio fischeri* (reviewed in [6], [7], [8], [9], [10]). This organism becomes luminescent when a high population density, representing a quorum, is reached within the light organ of the squid *Euprymna scolopes*. *V. fischeri* measures its population density by producing *N*-(3-oxo-hexanoyl)-L-homoserine lactone (oxoC6) using the LuxI enzyme [11]. Because the oxoC6 can freely diffuse across the bacterial cell wall, the accumulation of AHL indicates a high population density [12], [13]. The transcription factor LuxR binds oxoC6, dimerizes, and activates transcription of the *luxICDABE* operon resulting in luminescence [14], [15], [16]. LuxR/I systems have been found in numerous Gram-negative pathogens that colonize plants and animals and often regulate the pathogens' host interaction genes [17]. Presumably it is advantageous for the bacteria to delay the expression of genes that are likely to stimulate the host immune response until after a significant population density has been reached. The LuxI enzyme from a particular species often produces AHL(s) that differ from oxoC6 in the length of the acyl chain, the degree of saturation, or the modification at the 3-carbon position. The cognate LuxR homolog detects the specific AHL variant made by its partner LuxI enzyme.

The genera *Escherichia* and *Salmonella* encode a single LuxR homolog named SdiA but do not encode an AHL synthase [18], [19], [20], [21]. With some good fortune, the genes regulated by SdiA in *Salmonella enterica* serovar Typhimurium (hereafter referred to as *S.* Typhimurium) were identified without knowledge of the signal. Random MudJ insertions (which create *lacZY* transcriptional fusions) were isolated in a strain in which *sdiA* was conditionally expressed from a multicopy plasmid. Fusions that respond to plasmid-encoded *sdiA* were isolated in two loci, *srgE* and the *rck* operon [18], [19], [20]. Overexpression of *sdiA* had bypassed the requirement for AHL [19]. The fusions obtained were used to identify the signals required for activity of SdiA expressed from its natural position in the chromosome. However, this step was troublesome because the chromosomal transcriptional fusions respond to SdiA only under specific growth conditions [20]. For unknown reasons, plasmid-based fusions lack these environmental restraints. Therefore, plasmid-based fusions were used to identify the AHLs detected by *sdiA* under standard laboratory growth conditions [19]. SdiA was found to detect a wide range of AHLs at concentrations that are physiologically relevant [19], [20], [22], [23]. SdiA can detect *N*-(3-oxo-octanoyl)-L-homoserine lactone (oxoC8) at 1 nM and oxoC6 at 5 nM. At 50 nM, SdiA can detect oxoC10 as well as the unmodified variants *N*-hexanoyl-L-homoserine lactone (C6), and *N*-octanoyl-L-homoserine lactone (C8) [19], [21], [24]. Once the appropriate AHLs were identified, growth conditions were screened that would allow chromosomal fusions to respond to chromosomal *sdiA* and AHL [20]. Our work with a gene encoded adjacent to *sdiA*, named *sirA* (*uvrY* in *E. coli*) led to the discovery that growth in motility agar at 37°C allows optimal activity of both SdiA and SirA [20], [25], [26]. Like plasmid-based expression of *sdiA*, lowering the temperature to 30°C can cause *sdiA*-dependent activation in the absence of AHL [20]. However, this only occurs with the *srgE* locus because the *rck* operon is not expressed at temperatures below 37°C. The lowered temperature may allow SdiA to oligomerize in the absence of signal. The mechanism by which motility agar enhances expression of chromosomal fusions (but not plasmid-based fusions) is not known.

The identification of the SdiA regulon of *E. coli* has been even more problematic than that of *S.* Typhimurium. Three genetic screens identified *sdiA* as a gene that, when expressed from a plasmid, could give rise to a particular phenotype. These included suppression of a cellular division block [27], resistance to mitomycin C [28], and resistance to quinolones [29]. Plasmid-based expression of *sdiA* caused these phenotypes by upregulating the *ftsQAZ* or *acrAB* loci. Additionally, when *sdiA* is expressed from a plasmid in *E. coli*, the strain becomes up to seven-fold more resistant to fluoroquinolones (norfloxacin, ofloxacin, and ciprofloxacin) and chloramphenicol, and two-fold more resistant to kanamycin and tetracycline [29]. An *sdiA* null mutant strain was up to three-fold more sensitive to the fluoroquinolones, but not chloramphenicol, nalidixic acid or tetracycline [29]. This increase in resistance was hypothesized to be due, at least in part, to *sdiA* increasing the expression of the *acrAB* genes that encode a multidrug efflux pump. When cellular protein levels were measured, plasmid-based expression of *sdiA* led to a 4.3-fold increase in AcrA levels and 4.5-fold increase in AcrB levels [29]. A chromosomal *sdiA* mutation led to a 50% decrease in AcrB but no difference in AcrA protein levels compared to wild-type. It is interesting to note that the overexpression of *sdiA* in an *acrAB* mutant did not abolish the increase in fluoroquinolone resistance, suggesting that *sdiA* might affect another efflux pump or pathway to drug resistance [29]. AHL was not used in any of the assays.

A microarray study was performed to identify the *sdiA* regulon of *E. coli*. This study used plasmid-based expression of *sdiA* in the absence of AHL [30]. These experiments identified 75 genes that were up-regulated and 62 genes that were down-regulated in response to plasmid-based expression of *sdiA*. The *ftsQAZ* and *acrAB* loci were among the genes identified, again confirming that these loci respond to plasmid-based expression of *sdiA*. The microarray study and a second independent study also showed that the *uvrY* gene (*sirA* in *Salmonella*) is regulated by plasmid-encoded *sdiA* [30], [31]. In Enterohaemorrhagic *E. coli* O157:H7 (EHEC), it was determined that the expression of EspD, intimin, and flagellar proteins were reduced by plasmid-based expression of *sdiA* [32]. The microarray study with *E. coli* K-12 also observed repression of flagellar genes by plasmid-based expression of *sdiA* [30]. However, for all of the genes discussed above, the effect of chromosomal *sdiA* was either not reported or was found to be less than two-fold.

We hypothesized that overexpression of *sdiA* causes a pleiotropic effect in *E. coli* that does not occur in *S.* Typhimurium [21]. Furthermore, we know that *sdiA* of *E. coli* is expressed from the chromosome and functional because it activates a plasmid-based *srgE-luxCDABE* fusion from *S.* Typhimurium in response to AHLs [21]. Recently, Van Houdt *et. al.*, performed a genetic screen with *E. coli* in which 13,100 plasmid-based fusions were screened for a response to AHL during growth in LB broth at 30°C [33]. Six up-regulated and nine down-regulated promoters were identified and confirmed to be dependent upon *sdiA* expressed from its natural position in the chromosome. Interestingly, *uvrY* was the only gene that overlapped between this set of genes and the set derived from *sdiA* overexpression studies. The fold-induction or repression observed in this chromosomal *sdiA* based study was never more than 1.5-fold for any of the genes [33]. Additionally, a second microarray study was performed recently that compared wild-type *E. coli* to *sdiA* mutant *E. coli* in late stationary phase at 30°C, although AHL was not included in the growth medium [34]. Forty genes were repressed by *sdiA* and 42 were activated. Except for the repression of flagellar genes, the vast majority of the genes identified in this study are different than those found in previous studies.

In this report, we examined the regulation of two loci, *ftsQAZ* and *acrAB*, that were previously determined to respond to plasmid-based expression of *sdiA*. We tested the hypothesis that these genes are regulated by *sdiA* expressed from its natural location in the chromosome if AHL is present. However, we observed no regulation of these genes in response to chromosomal *sdiA* and AHL. Therefore, we decided to perform a new genetic screen in *E. coli* using the information gained from our studies of the *sdiA* regulon of *S.* Typhimurium. We used a transposon to create chromosomal fusions in a wild-type background (in which *sdiA* is expressed from its natural position in the chromosome) and then screened the fusions for a response to AHL during growth in motility agar at 37°C. We performed this screen with both *E. coli* K-12 and EHEC. The AHL-responsive fusions were then tested for *sdiA*-dependence.

## Results

### Quinolone Resistance Is Not Increased by Chromosomal *sdiA* and/or AHL

**Figure 1:**
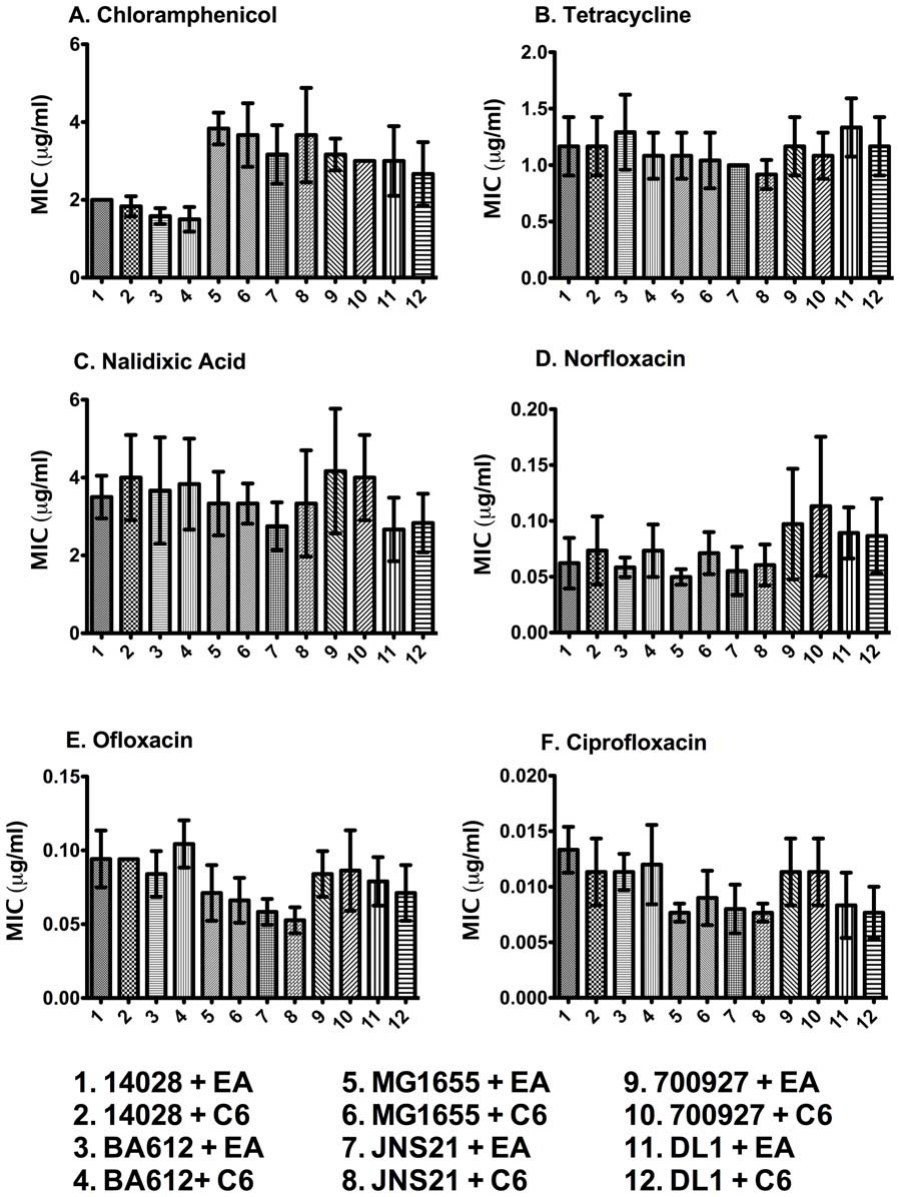
Antibiotic resistance of *S.* Typhimurium, *E. coli* K-12, and EHEC as measured by E-Test strips. The graphs show the MIC of the wild-type strains and their respective isogenic *sdiA* mutants (BA612, JNS21, and DL1, respectively). Each bar is the average of two separate experiments performed in triplicate and error bars represent standard deviation.

It has been reported that plasmid-based expression of *sdiA* causes an increase in quinolone resistance in *E. coli* [29]. In this report, we tested the hypothesis that *sdiA* expressed from its natural position in the chromosome can increase resistance to quinolones in response to AHL. We used two assays to measure antibiotic resistance. The first was the E-Test strip assay, which utilizes a plastic strip that is coated with a gradient of antibiotic. Bacteria are spread on the surface of an agar plate and then the strip is placed on the plate. The minimum inhibitory concentration (MIC) is read from where the zone of growth inhibition intersects the strip. Using the E-Test strips, neither *sdiA* nor AHL had any effect on the MIC of *S.* Typhimurium, *E. coli* K-12, or EHEC for chloramphenicol, tetracycline, nalidixic acid, norfloxacin, ofloxacin, or ciprofloxacin (Figure 1). The strains used are described in Table 1.

**Table 1:** Strains and Plasmids.

Strain or Plasmid	Genotype	Source or Reference
**Strains**		
14028	Wild-type *Salmonella enterica* serovar Typhimurium	ATCC
700927	Wild-type *Escherichia coli* O157:H7 (EHEC)	ATCC
AL4001	BA4000 *gadW4001*::mTn*5luxkan2*	This study
BA612	14028 *sdiA1*::mTn*3*	[18]
BA4000	nal resistant mutant of BW25113	This study
BW20767	*E. coli leu-63*::IS*10 recA1 creC510 hsdR17 endA1 zbf-5 uidA*(ΔMluI)::*pir* ^+^ *thi* RP4-2-tet::Mu-1kan::Tn*7*	[44]
BW25113	Δ(*araD-araB*)567 Δ*lacZ4787*(::*rrnB*-3) *lacIp*-4000(*lacIQ*) *rph*-1 Δ(*rhaD*-*rhaB*)568 *hsdR514*	[43]
DL1	700927 *sdiA25*::EZ-Tn*5*<kan-2>	This study
JLD271	WM54 *sdiA271*::cam	[42]
JLD370	WM54 *acrA*+/*acrA::lacZYA* integrant	This study
JLD373	JLD370 *sdiA271*::cam	This study
JLD404	nal resistant mutant of 700927	This study
JLD604	JLD404 *fliE604*::mTn*5luxkan2*	This study
JLD605	JLD404 *gadE605*::mTn*5luxkan2*	This study
JLD607	JLD404 *yhiD607*::mTn*5luxkan2*	This study
JLD610	JLD404 *hdeA608*::mTn*5luxkan2*	This study
JLD800	AL4001 *sdiA271*::cam	This study
JLD803	JLD604 *sdiA*::pRE112	This study
JLD804	JLD605 *sdiA*::pRE112	This study
JLD806	JLD607 *sdiA*::pRE112	This study
JLD809	JLD610 *sdiA*::pRE112	This study
JLD3000	MG1655 *ftsZ* ^+^ *-*FRT-*cam*-FRT	This study
JLD3004	WM54 *ftsZ* ^+^ *-*FRT-*cam*-FRT	This study
JLD3011	WM54 *ftsZ* ^+^-*lacZYA*	This study
JLD3013	JLD3011 *sdiA271*::cam	This study
JNS21	MG1655 *sdiA25*::EZ-Tn*5*<kan-2>	This study
MG1655	Wild-type *Escherichia coli* K-12	*E. coli* Genetic Stock Center
S17λpir	*E. coli recA pro hsdR* <RP4-2-*tet*::Mu-1*kan*::Tn*7*> λpir	[45]
WM54	*E. coli* K-12 Δ*lacX74*	Bill Metcalf
**Plasmids**		
pBAD33	Arabinose-conditional expression vector, p15A (Cam^r^)	[46]
pCE36	FRT *lacZY+ t_his_ ori* R6K (Kan^r^)	[47]
pCLF3	FRT*-cam-*FRT oriR6K (Amp^r^)	[48]
pCP20	*cI857* λPR *flp* pSC101 oriTS (Amp^r^ Cam^r^)	[49]
pCX16	pGB2 carrying *E. coli sdiA* (Spec^r^)	[27]
pGB2	pSC101 cloning vector (Spec^r^)	[27]
pKD46	PBAD *gam bet exo* pSC101 oriTS (Amp^r^)	[43]
pJLD1203	pSB401 *fliE-luxCDABE* (Tet^r^)	This study
pJLD1505	*acrA-lacZYA* fusion in pVIK112 (Kan^r^)	This study
pJLD2000	Central portion of *sdiA* in pRE112 (Cam^r^)	This study
pJVR2	*sdiA* under control of *araBAD* promoter; pACYC origin (Cam^r^)	[18]
pRE112	Suicide vector, *sacB, ori* R6K (Cam^r^)	[50]
pSB401	*luxR* ^+^ *luxI*::*luxCDABE*; pACYC origin (Tet^r^)	[51]
pUT mTn5 lux kan2	Suicide vector, *ori* R6K, mini-Tn*5* Km2 *luxCDABE* transposon, *mob* ^+^ (RP4) (Amp^r^ Kan^r^)	[41]
pVIK112	*lacZYA* transcriptional fusion vector, *ori* R6K (Kan^r^)	[52]

**Figure 2:**
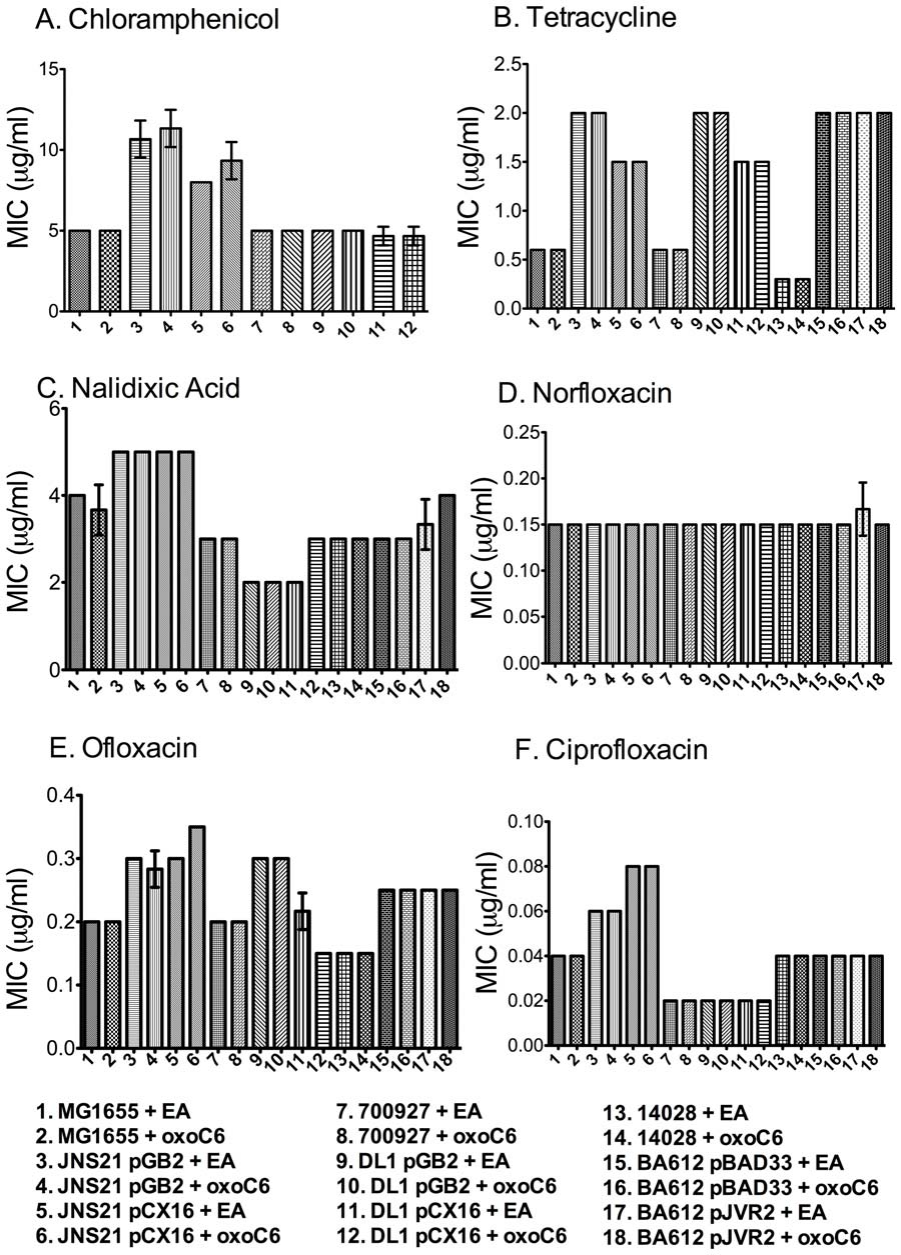
Antibiotic resistance of *E. coli* K-12, EHEC and *S.* Typhimurium grown in motility agar at 37°C. Strains were grown in LB 0.3% motility agar with either 1 µM oxoC6 or 0.1% EA and a dilution series of each antibiotic tested. The minimum inhibitory concentration was read from the well in which no visible growth was seen at the inoculation point. In panel A, *S.* Typhimurium was not tested because the *sdiA* plasmid carries a gene for chloramphenicol resistance. Each strain was assayed in triplicate and error bars represent standard deviation.

**Figure 3:**
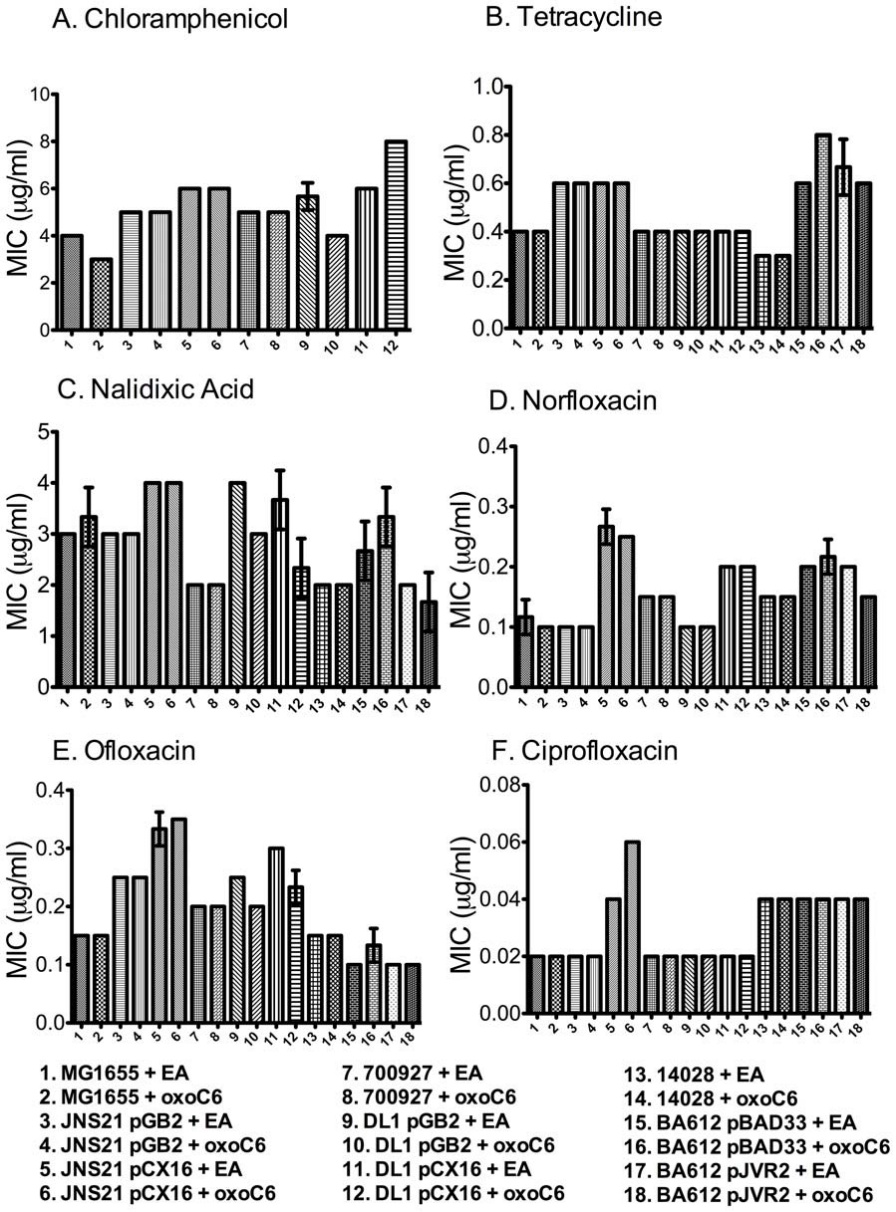
Antibiotic resistance of *E. coli* K-12, EHEC and *S.* Typhimurium grown in motility agar at 30°C. Strains were grown in LB 0.3% motility agar with either 1 µM oxoC6 or 0.1% EA and a dilution series of each antibiotic tested. The minimum inhibitory concentration was read from the well in which no visible growth was seen at the inoculation point. In panel A, *S.* Typhimurium was not tested because the *sdiA* plasmid carries a gene for chloramphenicol resistance. Each strain was assayed in triplicate and error bars represent standard deviation.

Based on our previous observation that SdiA of *S.* Typhimurium appears to be most active in motility agar [20], we tested the hypothesis that *sdiA* would be involved in antibiotic resistance during growth in motility agar. A dilution series of each antibiotic was added to molten motility agar at 55°C. The motility agar was dispensed into the wells of 96-well plates and allowed to cool to room temperature. The various bacterial strains were then inoculated into the motility agar by stabbing the center of each well. The MICs were determined in the presence and absence of AHLs. We also included two variables that have made a difference in past publications, i.e., plasmid-based expression of *sdiA* versus chromosomal expression of *sdiA*, and growth at 30°C versus 37°C. In *E. coli* K-12, EHEC, and *S.* Typhimurium we observed no AHL-dependent increase in antibiotic resistance at either temperature (Figures 2 and 3). However, using plasmid-encoded *sdiA* we did observe 2-fold changes in response to some antibiotics. Some of these effects appeared to be partially or completely due to the vector used to encode *sdiA*, while other effects were due to *sdiA* and not the vector (Figures 2 and 3). Thus, we have confirmed the previously published results that plasmid-encoded *sdiA* can lead to small changes in antibiotic resistance but we observe no effect of AHL or *sdiA* on antibiotic resistance when *sdiA* is expressed from its natural position in the chromosome.

### The Expression of *acrAB* Is Not Increased by Chromosomal *sdiA* and/or AHL

**Figure 4:**
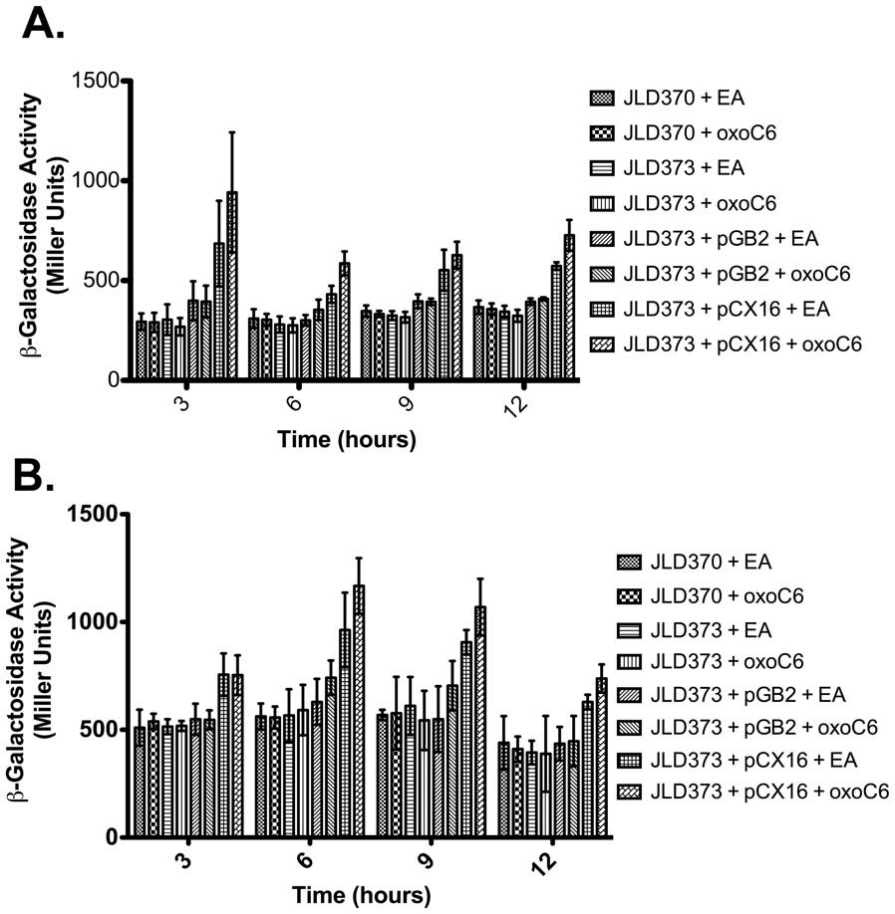
Regulation of *acrA* by *sdiA*. A chromosomal merodiploid *acrA* ^+^/*acrA*-*lacZY* fusion was constructed in a Δ*lac* mutant *E. coli* strain (JLD370), and in an isogenic *sdiA* mutant (JLD373). Additionally, derivatives of the *sdiA* mutant were constructed that contained either a low copy number vector expressing *sdiA* (pCX16) or the vector control (pGB2). The strains were subcultured 1:100 into LB broth containing either 1 µM oxoC6 or EA. The cultures were incubated with shaking at 30°C (A) and 37°C (B). Samples were removed from the cultures at time points for β-galactosidase assays. Each strain was assayed in triplicate and error bars represent standard deviation. * denotes p<0.05 compared to the adjacent solvent control.

When expressed from a plasmid, *sdiA* has been shown to increase the expression of the *acrAB* locus in *E. coli* K-12 [29], [30]. To test the hypothesis that *acrAB* can respond to chromosomal *sdiA* and AHL, we constructed a chromosomal merodiploid *acrA* ^+^/*acrA*::*lacZY* fusion in *E. coli* K-12 and an isogenic *sdiA* mutant and grew them in the presence of AHL or EA. As seen in Figure 4 there was no significant difference in β-galactosidase activity between the wild-type and *sdiA* mutant strains at either 30°C or 37°C. However, when *sdiA* was expressed from a plasmid we observed an increase of up to two-fold in *acrA* expression compared to the vector control (Figure 4), confirming the previously published results [29], [30]. AHL slightly increased the activity of plasmid-encoded *sdiA* (Figure 4).

### The Expression of *ftsQAZ* Is Not Increased by Chromosomal *sdiA* and/or AHL

**Figure 5:**
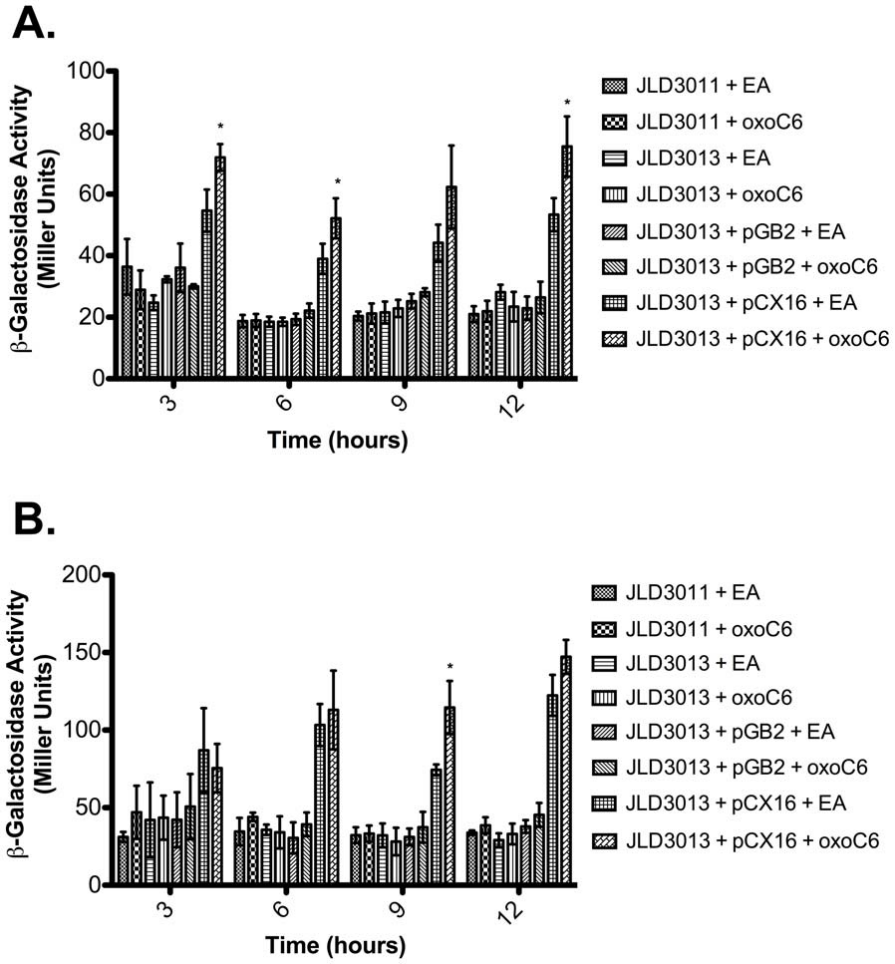
Regulation of *ftsQAZ* by *sdiA*. A chromosomal *ftsQAZ*-*lacZ* fusion was constructed in a Δ*lac* mutant *E. coli* strain (JLD3011), and an isogenic *sdiA* mutant (JLD3013). Additionally, derivatives of the *sdiA* mutant were constructed that contained either a low copy number vector expressing *sdiA* (pCX16) or the vector control (pGB2). The strains were subcultured 1∶100 into LB broth containing either 1 µM oxoC6 or EA. The cultures were incubated with shaking at 30°C (A) and at 37°C (B). Samples were removed from the cultures at time points for β-galactosidase assays. Each strain was assayed in triplicate and error bars represent standard deviation. * denotes p<0.05 compared to the adjacent solvent control.

The *ftsQAZ* operon has an essential role in cell division. Therefore, we made a chromosomal *lacZY* transcriptional fusion immediately after the stop codon of *ftsZ* but before the transcription terminator (see Materials and Methods). Using this construct we were able to confirm the previously published observation that expression of *sdiA* from a plasmid increases *ftsQAZ* expression by up to four-fold (Figure 5). However, when *sdiA* is expressed from its natural position in the chromosome it has no effect on *ftsQAZ*, even in the presence of AHL at either 30°C or 37°C (Figure 5).

### Identification of AHL-Responsive Transcriptional Fusions in *E. coli* K-12 and EHEC

To identify genes that are regulated in response to AHLs in *E. coli* K-12 and EHEC we constructed random mTn*5* transposon mutants, as described in Materials and Methods. The transposon creates a transcriptional luciferase (*luxCDABE*) fusion upon insertion. Each mutant was screened for AHL-responsive luciferase activity by patching into two adjacent wells of a 96-well plate filled with motility agar containing either AHL (oxoC6) or the solvent control, acidified ethyl acetate (EA). The plates were incubated at 37°C and luminescence readings of each well were recorded after 9 hours. We screened 10,000 *E. coli* K-12 mutants and 10,000 EHEC mutants. After the initial screening, candidate mutants were tested again for AHL responsiveness in triplicate using a more precise procedure in which molten motility agar is seeded with a liquid overnight culture of the mutant in question, rather than stabbing the mutant into the wells (see Materials and Methods).

**Figure 6:**
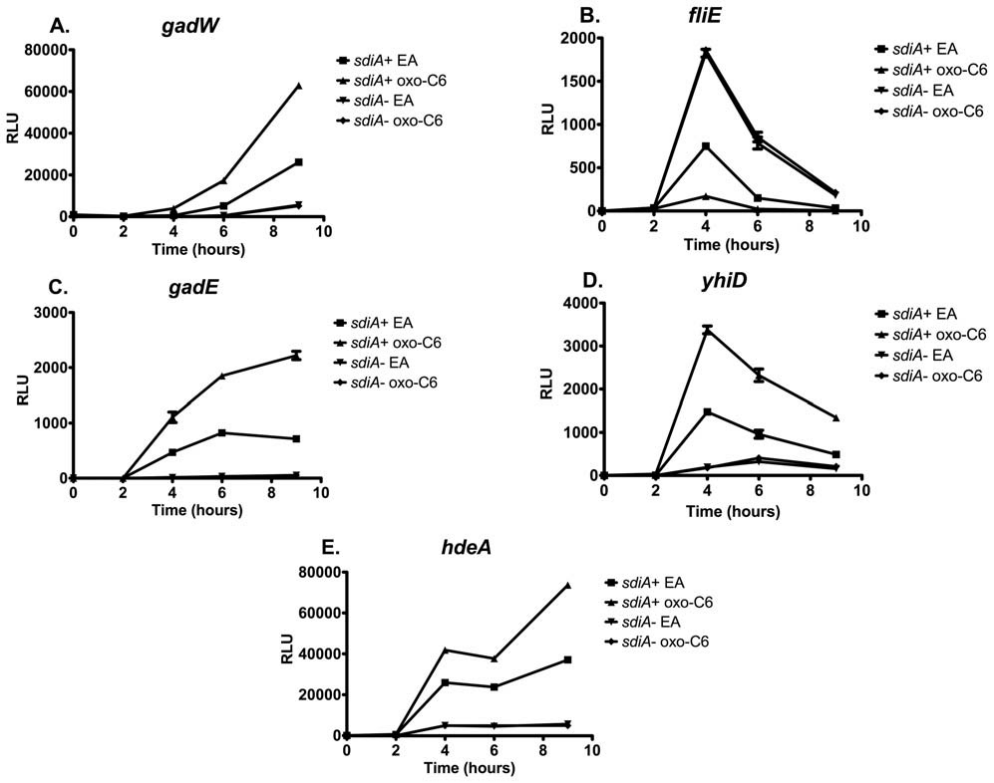
Regulation of AHL-regulated genes of *E. coli* K-12 and EHEC in motility agar containing either 100 nM oxo-C6 or the solvent control, EA at 37°C. Luminescence in relative light units (RLU) was measured using a Wallac Victor^2^ 1420 multimode plate reader at the time intervals noted. Each strain was assayed in triplicate and error bars represent standard deviation. A) AL4001*/*JLD800 (*gadW*), B) JLD604/JLD803 (*fliE*), C) JLD605/JLD804 (*gadE*), D) JLD607/JLD806 (*yhiD*), E) JLD610/JLD809 (*hdeA*).

**Figure 7:**
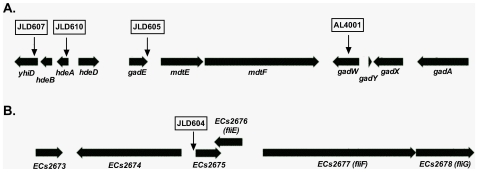
A) Acid fitness island of *E. coli*. The transposon insertion in *E. coli* K-12, AL4001, is within *gadW* at nucleotide 3662317 of Genbank accession number U00096. The transposon insertions in the EHEC strains are shown on the same map but the nucleotide positions are from Genbank accession number BA000007. JLD605 is within *gadE* at nucleotide 4401036; JLD607 is within *yhiD* at nucleotide 4397949; JLD610 is within *hdeA* at nucleotide 4398821. B) JLD604 is just upstream of ECs2675 in the anti-sense orientation at nucleotide 3662317.

One mutant in *E. coli* K-12 and four in EHEC showed a consistent change in luciferase expression in the presence of oxoC6 compared to the solvent control, EA. One of the fusions was down-regulated 7-fold by oxoC6 while four were up-regulated by up to 7-fold (Figure 6). The DNA sequence of each transposon insertion site was determined using the mutant chromosomal DNA as the template and two different sequencing primers that bind within the transposon sequence (primers are listed in Table 2). The two sequencing reactions yielded the same result in each case with regard to the transposon insertion point (Figure 7). The single AHL-regulated fusion in *E. coli* K-12 (AL4001) was inserted in the *gadW* gene. In EHEC there were three transposon-based fusions that were up-regulated in response to AHL. JLD605 contained an insertion in ECs4392 (ortholog of *E. coli* K-12 *gadE/yhiE*); JLD607 contained an insertion in ECs4388 (ortholog of *E. coli* K-12 *yhiD*); and JLD610 contained an insertion in ECs4390 (ortholog of *E. coli* K-12 *hdeA*). The one fusion in EHEC that was down-regulated, JLD604, was inserted just upstream of ECs2675, encoding a hypothetical protein, but the transposon orientation was anti-sense suggesting that expression was being driven from the ECs2676 gene promoter (ortholog of *E. coli* K-12 *fliE*). Because of the unusual location of the fusion in JLD604, we cloned the *fliE* promoter region into pSB401 to form a *fliE-luxCDABE* transcriptional fusion. When this construct, pJLD1203, was placed into wild-type and *sdiA* mutant *E. coli*, we observed that the *fliE* promoter is indeed repressed by *sdiA* (data not shown).

**Table 2:** Oligonucleotides used.

Oligo	Sequence	Description
BA184	GATGTGCTGCAAGGCGATTAAGTTG	For sequencing *lacZ* fusion junctions
BA247	GAGTCATTCAATATTGGCAGGTAAACAC	For sequencing mTn*5luxkan2* insertion sites (anneals to *luxC*)
BA408	GCGATGGCGGGGCTGATTGACGGTA	3′ oligo for amplifying *E. coli yecC-sdiA* region
BA409	GGTCACGCCCGTCACCAACGGCTTA	5′ oligo for amplifying *E. coli yecC-sdiA* region
BA502 (C1)	TTATACGCAAGGCGACAAGG	binds within FRT-cam-FRT
BA1090	GAATGTATGTCCTGCGTCTTGAGTA	For sequencing mTn*5luxkan2* insertion sites (anneals to *luxC*)
BA1168	ATCCCAGCATTCCTGCGTAAGCAAGCTGATTAAGTGTAGGCTGGAGCTGCTTC	5′ primer for making the FRT site at the end of *ftsZ*
BA1505	GCCGGGTTCACCGAGAGTGAATTTT	For amplifying the *fliE* promoter region of EHEC
BA1506	AATTGCGCCACCATCCTGATCGGAA	For amplifying the *fliE* promoter region of EHEC
BA1533	TCTAGAGCGCTTGCTGAAATTCATCTCTGGC	For amplifying internal fragment of *sdiA* in EHEC with *xbaI* site
BA1534	CCCGGGGGCTTTCTACACCAATTACCCTGAG	For amplifying internal fragment of *sdiA* in EHEC with *smaI* site
BA1817	GAGCCTCGAAACCCAAATTCCAGTCAATTCCATATGAATATCCTCCTTAG	3′ primer for making the FRT site at the end of *ftsZ*
BA1818	ACCAGTCGTCGCTGAAGTGGCAAAAGATTT	binds within *ftsZ* pointing downstream

### AHL Responses Are *sdiA*-Dependent

We hypothesized that the AHL-responsiveness of each fusion was dependent upon the *sdiA* gene. To test this, an isogenic *sdiA* mutation was placed into each of the fusion strains, as described in Materials and Methods. As expected, the response of each fusion to AHL in motility agar was eliminated by the *sdiA* mutation (Figure 6). Interestingly, there was a substantial amount of AHL-independent SdiA activity observed with each fusion. Therefore, in *E. coli* K-12 and EHEC, SdiA is partially active even in the absence of AHL. This has been observed previously with the *srgE* gene of *Salmonella*, but only at the lower temperature of 30°C [20]. Previous reports have observed that *sdiA*-regulated fusions in *E. coli* K-12 are more responsive at 30°C than at 37°C [33], [34].

**Figure 8:**
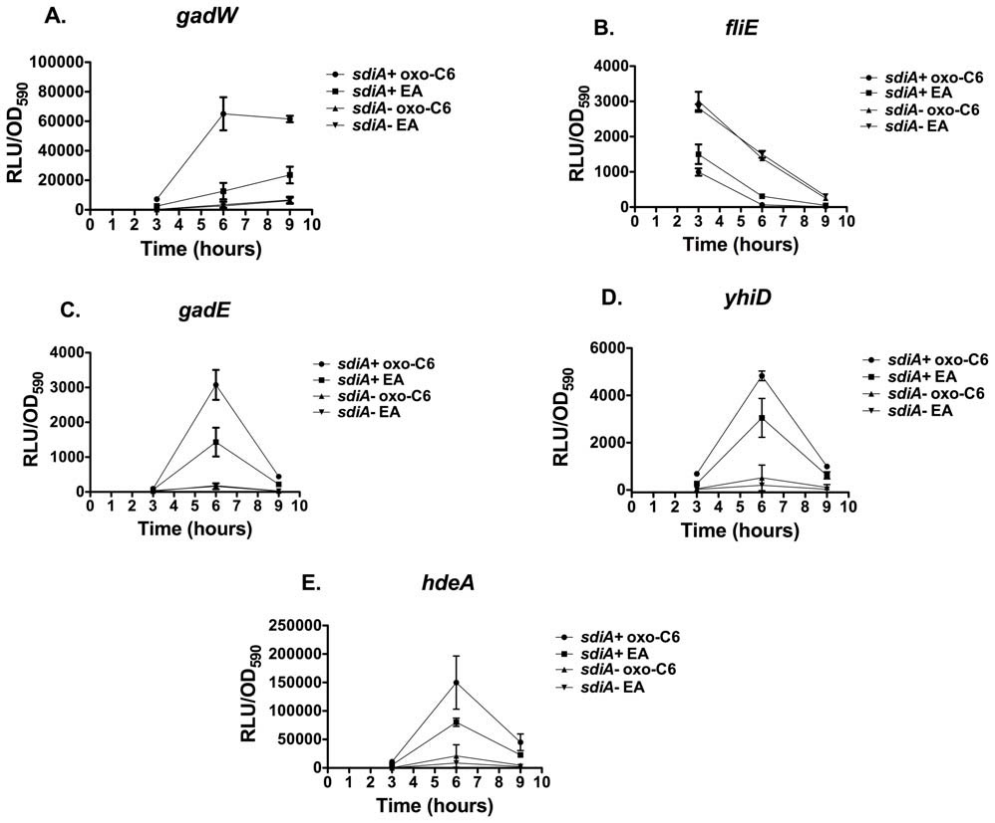
Regulation of AHL-regulated genes of *E. coli* K-12 and EHEC in liquid cultures at 37°C containing either 1 µM oxo-C6 or the solvent control, EA. Luminescence in relative light units (RLU) and OD590 were measured using a Wallac Victor^2^ 1420 multimode plate reader at the time intervals noted. Each strain was assayed in triplicate and error bars represent standard deviation. A) AL4001*/*JLD800 (*gadW*), B) JLD604/JLD803(*fliE*), C) JLD605/JLD804 (*gadE*), D) JLD607/JLD806 (*yhiD*), E) JLD610 /JLD809 (*hdeA*).

**Figure 9:**
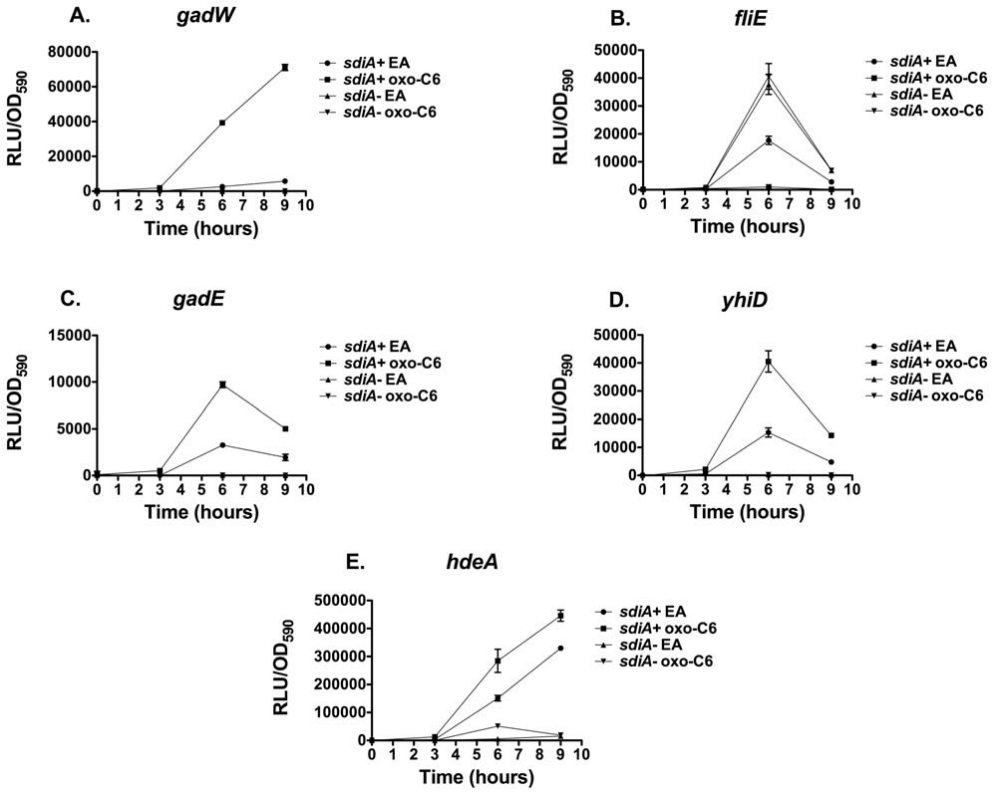
Regulation of AHL-regulated genes of *E. coli* K-12 and EHEC in liquid cultures at 30°C containing either 1 µM oxo-C6 or the solvent control, EA. Luminescence in relative light units (RLU) and OD590 were measured using a Wallac Victor^2^ 1420 multimode plate reader at the time intervals noted. Each strain was assayed in triplicate and error bars represent standard deviation. A) AL4001*/*JLD800 (*gadW*), B) JLD604/JLD803 (*fliE*), C) JLD605/JLD804 (*gadE*), D) JLD607/JLD806 (*yhiD*), E) JLD610 /JLD809 (*hdeA*).

To determine if temperature affects the *E. coli* K-12 and EHEC *sdiA*-regulated fusions, each strain was assayed in liquid broth at 37°C and 30°C (Figures 8 and 9, respectively). Expression in the wild-type background was several orders of magnitude higher than in the *sdiA* mutant background at both temperatures. However, much of this was AHL-independent. All fusions except for *gadW* demonstrated higher overall levels of expression at 30°C than at 37°C. The maximal fold-induction between EA and AHL was also greater at 30°C than at 37°C for all fusions. The *gadW* fusion is induced 6-fold by AHL at 37°C and 25-fold at 30°C. The *fliE* fusion is repressed 5-fold by AHL at 37°C and 18-fold at 30°C. The *gadE* fusion is only induced 2.2-fold by AHL at 37°C but is induced 16-fold at 30°C. The *yhiD* and *hdeA* fusions are the least regulated being induced 2 to 5-fold by AHL under all conditions. Interestingly, at 37°C there is no difference in AHL-dependent maximum fold-induction between growth in motility agar (Figure 6) or liquid broth (Figure 8). This is different than what is observed in *Salmonella*, where motility agar enhances AHL-dependent regulation [20]. However, two overall trends remained the same at the two temperatures: i) there is substantial basal activity of SdiA even in the absence of AHL; and ii) the response to AHL is completely dependent upon *sdiA*.

**Figure 10:**
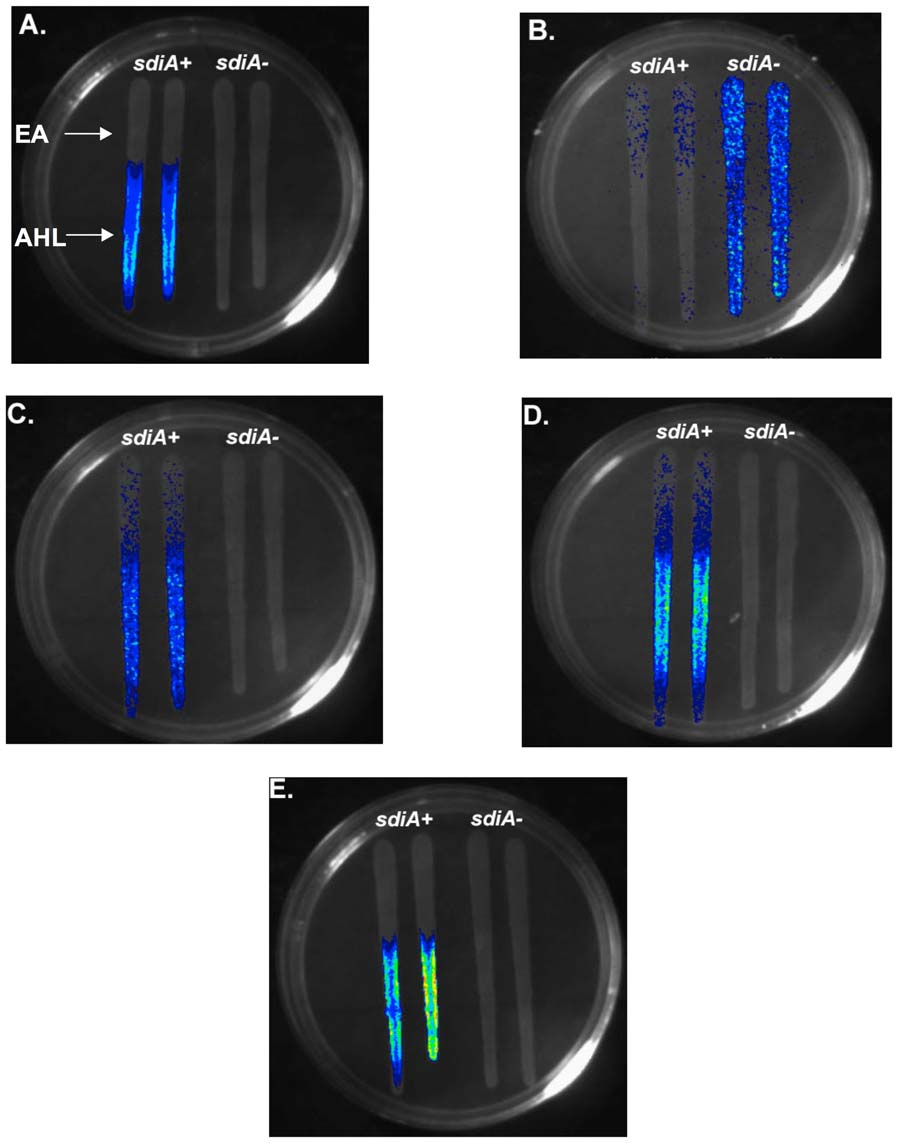
Cross streak assays of the *E. coli* K-12 and EHEC *lux* fusions. The chromosomal *lux* fusions and their respective *sdiA* mutants were grown in broth overnight. The strains were dripped down the plate perpendicular to 20 µl of EA then 20 µl of 10 µM oxoC6 (diagrammed in Panel A for all panels). Plates were incubated at 37°C for 7 hours then light emission was imaged using a C2400-32 intensified charge-coupled device camera with an Argus 20 image processor. A) AL4001*/*JLD800 (*gadW*), B) JLD604/JLD803 (*fliE*), C) JLD605/JLD804 (*gadE*), D) JLD607/JLD806 (*yhiD*), E) JLD610 /JLD809 (*hdeA*).

We also tested the fusions on solid LB agar using cross-streak assays (Figure 10). The fusions were clearly regulated by oxoC6 but not the solvent control EA. Four of the fusions were up-regulated in the presence of chromosomal *sdiA* (*gadW*, *gadE*, *yhiD* and *hdeA*), whereas the *fliE* promoter was down-regulated. This regulation was entirely dependent upon *sdiA*.

### Acid Resistance Phenotypes of *E. coli* K-12 and EHEC

**Figure 11:**
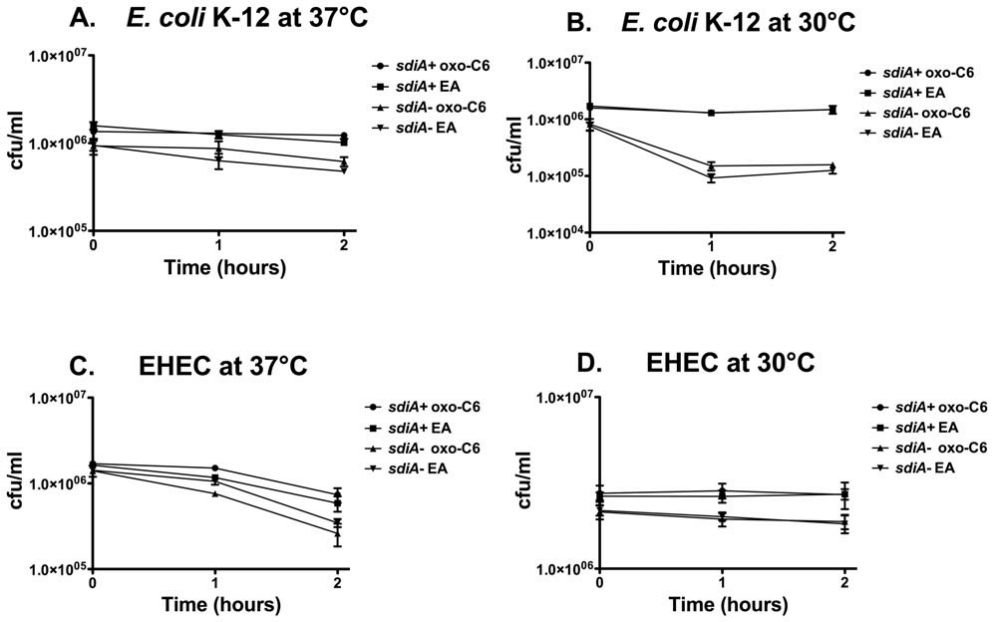
Acid resistance of *E. coli* K-12 and EHEC. Cells were grown in LB glucose with 1 µM oxo-C6 or 0.1% EA at either 37°C or 30°C and then subcultured into pre-warmed MEM with glucose and glutamate at pH 2.0 with continued incubation at the same temperature. Resistance to the acid challenge was determined by plating for cfu/ml every hour for two hours. *E. coli* K-12 wild-type MG1655 and *sdiA* mutant JNS21 at 37°C (A) and 30°C (B). EHEC wild-type 700927 and *sdiA* mutant DL1 at 37°C (C) and 30°C (D). Each strain was assayed in triplicate and error bars represent standard deviation.

The four up-regulated genes (*gadW*, *gadE*, *yhiD*, and *hdeA*) identified in our screen in *E. coli* K-12 and EHEC are known to be involved in the glutamate dependent acid resistance system (AR-2), and are located within the acid fitness island (AFI) (Figure 7). Interestingly, this island is not present in *Salmonella*. AR-2 uses a pair of glutamate decarboxylases (*gadA* and *gadB*) and an antiporter (*gadC*) to increase the pH of the cell [35], [36], [37]. The activation of four genes in the AFI led us to hypothesize that *sdiA* might enhance the glutamate dependent acid resistance phenotype in *E. coli* K-12 and EHEC. To test this hypothesis we performed acid resistance assays as previously described [37]. Wild-type and isogenic *sdiA* mutants of *E. coli* K-12 and EHEC were grown in LB broth with glucose to repress another acid resistance system (AR-1) and then sub-cultured into minimal E medium (MEM) with glucose and glutamate at pH 2.0 at either 30°C or 37°C. Cultures were sampled at zero, one, and two hours and plated for cfu. In *E. coli* K-12 at 30°C, the *sdiA* gene provided a 9-fold increase in survival (Figure 11b). A much smaller 2 to 3-fold survival phenotype was observed at 37°C and with EHEC (Figure 11). The addition of AHL to the growth and challenge media did not significantly increase the acid resistance phenotype, suggesting that the basal level of AHL-independent SdiA activity is sufficient for acid resistance.

## Discussion

Numerous studies have utilized plasmid-based expression of *sdiA* in order to study the SdiA regulon of *E. coli* [27], [28], [29], [30], [31], [32]. The rationale for using plasmid-encoded *sdiA* was that the AHL(s) that bind SdiA had not yet been discovered. In this report we addressed the question of whether the two most intensively studied loci identified in previous studies (*ftsQAZ* and *acrAB*) would respond to *sdiA* expressed from its natural position in the chromosome in the presence of AHL. Surprisingly, while we were able to replicate the observations that the genes respond to plasmid-encoded *sdiA*, the genes do not respond to chromosomal *sdiA* and/or AHL, at either 30°C or 37°C, in liquid broth or in motility agar. With regard to the antibiotic resistance phenotype, we were able to confirm small changes in antibiotic resistance when *sdiA* is expressed from a plasmid, but saw no differences in antibiotic resistance between a wild-type strain and an *sdiA* mutant of *E. coli* K-12, EHEC, or *S.* Typhimurium, at 30°C or 37°C, on solid agar or motility agar. Therefore we conclude that *ftsQAZ* and *acrAB* are not part of the *E. coli* SdiA regulon under the conditions tested.

Rather than continuing to individually test previously discovered genes for a response to chromosomal *sdiA* and AHL, we decided to start from the beginning and screen random transposon-based luciferase fusions for those that respond to AHLs. With this approach, both *sdiA* and the fusion are chromosomal during the screen. We screened 10,000 fusions in *E. coli* K-12 and 10,000 fusions in EHEC for a response to AHL. We identified *gadW* in *E. coli* K-12 and *gadE*, *yhiD* and *hdeA* in EHEC as being activated in response to AHL, and we found *fliE* in EHEC as being repressed in response to AHL. The response of these fusions to AHL was *sdiA*-dependent. The genes activated by *sdiA* suggest a role for SdiA in regulation of the glutamate dependent acid resistance system, AR-2. Surprisingly, this phenotype was independent of AHL even though the addition of AHL increases expression of the genes in the acid fitness island (AFI), suggesting that basal levels of SdiA activity are sufficient to increase acid resistance.

This is the third study that identified genes of the AFI as being regulated by chromosomally expressed *sdiA* [33], [34]. Oddly, the *gad* genes were not identified using plasmid-encoded *sdiA* and microarrays [30]. We determined that the *gadE* and *hdeA* promoters are responsive to plasmid-encoded *sdiA* (data not shown), so it is not clear why they were not identified in the microarray studies. However, both microarray studies identified numerous flagellar genes as repressed by plasmid-encoded *sdiA* [30], [34]. Additionally, flagellar gene expression is down-regulated by plasmid-encoded *sdiA* in EHEC [32]. The repression of our mTn*5luxkan2* fusion in *fliE* of EHEC confirms these observations by determining that *fliE* is repressed by chromosomal *sdiA*. As the EHEC strain tested here is not motile, we did not see any phenotype of this repression. It is also not clear why our screen failed to identify more flagellar genes as being repressed. We chose the conditions of our screen based on experience with *S.* Typhimurium where motility agar at 37°C is a good condition for observing *sdiA*-dependent activation of chromosomal fusions. In hindsight, SdiA of *E. coli* does not appear to be more active in motility agar than in liquid medium (compare Figure 6 to Figures 8 and 9). SdiA of *E. coli* also appears to be more active at 30°C than at 37°. Therefore, it might be worthwhile to take an iterative approach and repeat the screening process in liquid broth at 30°C.

It is quite possible that the SdiA regulon changes depending on the environmental or metabolic conditions. Thus, conditions may exist that allow chromosomal *sdiA* to activate the *acrAB*, *ftsQAZ*, or other genes. These conditions might remove barriers to individual target gene expression, or may increase SdiA expression or activity. Consistent with this possibility, the *sdiA* gene of *Salmonella* is upregulated during swarming motility [38]. However, until conditions are identified that allow chromosomal *sdiA* to activate a particular gene, that gene should not be considered to be a confirmed member of the *sdiA* regulon. It was recently discovered that *S.* Typhimurium SdiA becomes active in the Peyer's patches of mice infected with *Yersinia enterocolitica* [23]. *In vivo* environments like this may provide the most promising conditions for testing the *sdiA*-dependence of a particular gene. It will be interesting to determine if *E. coli* SdiA becomes active in a similar situation and to determine if any of the potential SdiA regulon members like *acrAB* and *ftsQAZ* become responsive to chromosomal *sdiA* in this setting. Furthermore, it will be of interest to determine the *in vivo* situation in which the *gad* genes and *fliE* are regulated by SdiA and play a role in the bacterium's fitness.

## Materials and Methods

### Bacterial Strains and Media

All bacterial strains and plasmids are listed in Table 1. Bacteria were grown in Luria-Bertani (LB) broth (EM Science). Agar was added to 0.3% (motility agar), or 1.2% (agar plates) as indicated. M9 minimal glucose medium was made as described previously [39]. When necessary, media were supplemented with appropriate antibiotics at the following concentrations (micrograms per milliliter): ampicillin, 100; kanamycin, 50; tetracycline, 20; nalidixic acid 75; and chloramphenicol, 30. *N*-hexanoyl-DL-homoserine lactone (C6) and *N*-(3-Oxohexanoyl)-L-homoserine lactone (oxoC6) (Sigma) were dissolved in acidified ethyl acetate (EA) and used at the concentrations noted in the text. EA is 0.1 ml glacial acetic acid per liter of ethyl acetate [40].

### Constructing and Screening Transposon Based Luciferase Fusions for AHL Responsiveness in EHEC and *E. coli* K-12

Transposon mutagenesis was performed by mating S17λpir+pUTmTn*5luxkan2* [41] with JLD404, a spontaneous nalidixic acid resistant mutant of EHEC strain ATCC 700927. The two strains were mated on an LB plate overnight at 37°C. The cells were then scraped from the agar, resuspended in LB broth, dilution plated onto LB agar containing kanamycin and nalidixic acid (100 µg/ml and 75 µg/ml, respectively) and incubated at 37°C overnight. Ten thousand of the resulting mutants were then stabbed individually into the wells of black 96 well plates containing 200 µl of LB 0.3% motility agar supplemented with either 100 nM oxoC6 or 0.01% EA and incubated at 37°C for 9 hours. Luminescence for each well was measured using the Wallac Victor^2^ plate reader (Perkin Elmer). Mutants that had a two-fold difference between EA and oxoC6 were struck to isolation on LB kan nal. Ten thousand mutants of *E. coli* K-12 strain BA4000 were constructed and screened in the same way, except that the initial screen was performed using 1 µM oxoC6 and 0.1% EA.

Mutants that had a two-fold difference or greater were inoculated into 100 µl of LB kan nal and grown at 37°C for 1 hour, shaking. 2.5 µl of the cultures were then used to inoculate 200 µl of LB motility agar containing 100 nM oxoC6 or 0.01% EA, in triplicate. Luminescence for each well was then measured using the Wallac Victor^2^ plate reader (Perkin Elmer).

### Cross Streak Assays

Mutants were grown overnight in LB kan nal at 37°C. The next day 20 µl of 10 µM oxoC6 and 20 µl of EA were dripped down the plate in separate locations and allowed to soak into the agar. 10 µl of each overnight culture was then dripped down the plate perpendicular to the EA and AHL cross streaks, in that order. The plates were then incubated at 37°C for 7 hours. Expression of luciferase by bacteria on plates was imaged and quantitated using a C2400-32 intensified charge-coupled device camera with an Argus 20 image processor (Hamamatsu Photonics).

### DNA Manipulation

Genomic DNA was isolated from overnight cultures of the mutants using the DNeasy Tissue Kit (Qiagen Inc., Valencia, CA). The transposon insertion site in the genomic DNA was sequenced twice, each time using a different primer that binds within the transposon. All primers are listed in Table 2. Both sequencing primers anneal to *luxC* of the transposon and are directed outward. Oligonucleotides were synthesized by Integrated DNA Technologies (IDT, Coralville, IA). DNA sequencing was performed by the Plant Microbe Genomics Facility at the Ohio State University.

### Construction of *sdiA* Mutants

A EZ-Tn5<kan-2> (Epicentre Biotechnologies) mutation was isolated in the *E. coli sdiA* gene. To do this the *yecC-sdiA* region was amplified using PCR with *Pfu* DNA Polymerase (Stratagene) and primers BA408 and BA409 with MG1655 as template. The PCR product was cloned into pCR-Blunt II-TOPO (Invitrogen). The *yecC-sdiA* fragment was removed from pCR-Blunt II-TOPO using *Xba*I and *Sst*I and cloned into pRE112 digested with the same enzymes resulting in pJS12. pJS12 was mutagenized in vitro with EZ-Tn5<kan-2> and transformed into S17λpir selecting LB kan. Location of EZ-Tn5<kan-2> inserts were determined using PCR screening followed by DNA sequencing. One isolate, pJS18, was saved for future use and contains EZ-Tn5<kan-2> after nucleotide 1994484 of Genbank accession number U00096.2 which is near the center of *sdiA*. S17λpir+pJS18 was mated with *E. coli* K-12 strain MG1655 and EHEC strain 700927 selecting on M9 glucose kan and screening for cam sensitive. The resulting *sdiA* mutants of *E. coli* K-12 and EHEC were named JNS21 and DL1, respectively. A second *sdiA* mutation in *E. coli* K-12 was also available, the *sdiA271*::cam mutation from JLD271 [42]. This mutation was moved into other *E. coli* K-12 strains using phage P1-mediated transduction.

Since EHEC does not have a transducing phage, we used single crossover disruptions of *sdiA* to quickly mutate the *sdiA* gene of the strains carrying AHL-regulated mTn*5luxkan2* fusions. A 378 bp internal fragment of the *sdiA* gene from EHEC, accession number BA000007, was amplified by PCR with an *XbaI* site at the 3′ end and a *SmaI* site at the 5′ end using *Taq* DNA polymerase and primers BA1533 and BA1534. The DNA fragment was then cloned with a TOPO TA cloning kit (Invitrogen). The *sdiA* fragment was digested out of pCR4-TOPO with *Xba*I and *Sma*I and ligated into pRE112 that had been cut with the same enzymes. The ligation was transformed into S17λpir cells. The resulting clones were screened by PCR for the presence and orientation of insert. One of the positive clones was selected for further use and named pJLD2000. The EHEC mTn*5luxkan2* mutants were mated with S17λpir+pJLD2000 and plated on M9 glucose kan cam.

### Liquid Assays for *lux* Fusions

LB kan cultures for the *sdiA*+ strains or LB kan cam cultures for the *sdiA* mutant strains were grown shaking at 37°C or 30°C overnight. They were then subcultured 1∶100 in triplicate into LB kan with either 1 µM oxoC6 or the appropriate volume of EA (0.1%) as a solvent control. They were grown with shaking at 37°C or 30°C and at time points 200 µl from each culture was placed in a black clear bottom 96 well plate. Both the OD_590_ and the luminescence were measured using a Wallac Victor^3^ plate reader.

### E-Test Strip Assays

The minimum inhibitory concentration of each antibiotic was measured according to manufacturer's instructions (AB bioMérieux). The strains were grown on an LB agar plate at 37°C with 1 µM C6 or without AHL (EA). Strains were then diluted in 0.85% NaCl solution, to an OD_550_ of 0.55 to 0.6. Using a sterile cotton tip applicator the strains were spread onto LB+EA and LB+AHL plates. After drying for 5 minutes, the E-Test strip was applied to the plate. The plates were then incubated at 37°C for 18 hours and read according to manufacturer's instructions. The antibiotics tested were chloramphenicol, ciprofloxacin, nalidixic acid, norfloxacin, ofloxacin and tetracycline.

### Motility Agar Antibiotic Resistance Assays

Strains were grown overnight in either LB broth with oxoC6 or LB broth with EA. 10 µl of each culture was mixed with 140 µl of LB motility agar (0.3% agar) containing either 1 µM oxoC6 or 0.1% EA, placed into the well of a 96 well plate, and incubated overnight at 37°C. This is the overnight growth plate. In a separate 96 well plate, 10 µl of an antibiotic dilution series was mixed with 140 µl of LB motility agar containing either 1 µM oxoC6 or 0.1% EA. This is the antibiotic plate. The antibiotic plate was inoculated with bacteria from the overnight growth plate by stabbing the wells of the overnight growth plate with a sterile toothpick and then stabbing the antibiotic plate. The antibiotic plates were incubated overnight at 30°C or 37°C. The MIC was read as the concentration where the strain showed no visible growth from the stab mark. Each strain was assayed in triplicate. The antibiotics tested were chloramphenicol, nalidixic acid, norfloxacin, ofloxacin, ciprofloxacin and tetracycline.

### Construction of *acrA* ^+^/*acrA*-*lacZY* Reporter Strains

The intergenic region between *acrA* and *acrR* from MG1655 was amplified by PCR with primers BA537 and BA538. The PCR product spans nucleotides 484610 to 485235 of Genbank accession number AE000155. The promoter region was then cloned into pCR-Blunt II-TOPO (Invitrogen). The promoter region was removed from the TOPO cloning vector by digesting with *EcoRI* and cloned into pVIK112 also digested with *EcoRI*. The resulting clones were then screened for insert and orientation by PCR. The correct construct, pJLD1505, was then transformed into BW20767. BW20767+pJLD1505 was mated with WM54 on an LB plate at 37°C overnight. The cells were resuspended and plated on M9+glucose kanamycin X-gal and incubated at 37°C for 48 hours. The resulting transconjugants were struck to isolation on M9+glucose kan and then screened for insertion of the suicide vector in the chromosome by PCR. One isolate was named JLD370 and saved for further use. An isogenic *sdiA* mutant was made by transducing the *sdiA271*::cam from JLD271 into JLD370 with P1 phage. The resulting strain was named JLD373.

### Construction of the *ftsZ-lacZ* Fusion

Primers were designed to match the end of *ftsZ*, including the stop codon, but before the transcriptional terminator. These primers, BA1168 and BA1817, also contained the P1 and P2 priming sites for pCLF3, respectively. Using these primers a cassette containing a chloramphenicol resistance gene flanked by regions of sequence identity to *ftsZ* was amplified from pCLF3 using *Taq* DNA polymerase, agarose gel purified (Qiagen gel extraction kit) and electroporated into arabinose induced MG1655+pKD46 cells [43]. The resulting colonies were screened for the proper insertion using PCR with primers C1 and BA1818. One isolate was saved for further use and named JLD3000. The *ftsZ*-FRT-*cam*-FRT mutation was moved into WM54 using P1 transduction, resulting in JLD3004. The chloramphenicol resistance gene was removed by transforming JLD3004 with pCP20 encoding Flp recombinase selecting on LB amp at 30°C. Colonies were struck to isolation and screened for chloramphenicol sensitivity at 30°C. Chloramphenicol sensitive colonies were electroporated with pCE36 selecting for Flp-dependent integration into the *ftsZ*-FRT site with selection on LB kan. pCP20 was eliminated by growth at 42°C and the resulting colonies were screened for the presence of the *ftsZ-lacZ* fusion using PCR with primers BA184 and BA1818. JLD3011 was then transduced with the *sdiA271*::*cam* mutation using phage P1 to give strain JLD3013.

### Construction of pJLD1203

The *fliE* promoter region of 700927 was amplified with Vent DNA Polymerase (NEB) and primers BA1505 and BA1506 and cloned into pCR-Blunt II-TOPO (Invitrogen). The *fliE* insert was removed from pCR-Blunt II-TOPO using *Eco*RI and ligated into pSB401 that had been cut with *Eco*RI as well (removing the *luxRI*' insert and replacing with *fliE*). The presence and orientation of insert were confirmed by PCR.

### Beta-Galactosidase Assays

Strains were grown overnight in LB broth and then subcultured 1∶100 into LB broth containing either 1 µM oxoC6 or 0.1% EA. The cultures were grown with shaking at 30°C or 37°C. At time points, β-galactosidase activity was assayed as previously described using ONPG as substrate [39].

### Acid Resistance Assays

Acid resistance assays were performed as described previously [37]. Cells were grown overnight in LB broth supplemented with 0.4% glucose and either 1 µM oxoC6 or 0.1% EA at either 37°C or 30°C. The overnight stationary phase cultures were diluted 1∶1000 into pre-warmed minimal E medium with 0.4% glucose (MEM+G) (pH 2.0) supplemented with 1.6 mM glutamate and either 1 µM oxoC6 or 0.1% EA. Viable cell counts were determined by dilution plating at 0, 1, and 2 hours after the acid challenge.
